# Fecal Microbiota Transplantation as Therapy for Treatment of Active Ulcerative Colitis: A Systematic Review and Meta-Analysis

**DOI:** 10.1155/2021/6612970

**Published:** 2021-04-23

**Authors:** Xiaolei Liu, Yan Li, Kaichun Wu, Yongquan Shi, Min Chen

**Affiliations:** ^1^Department of Medical Insurance, Xijing Hospital, Air Force Medical University, Xi'an, Shaanxi Province 710032, China; ^2^Department of Gastroenterology, The Affiliated Hospital of Qinghai University, Xining, Qinghai Province 810001, China; ^3^State Key Laboratory of Cancer Biology, National Clinical Research Center for Digestive Diseases and Xijing Hospital of Digestive Diseases, Air Force Medical University, Xi'an, Shaanxi Province 710032, China

## Abstract

**Aim:**

Increasing evidence supports the role of the gut microbiota in the etiology of ulcerative colitis (UC). Fecal microbiota transplantation (FMT) is a highly effective treatment against recurrent Clostridium difficile infection; however, its efficacy in UC is still controversial. A systematic review and meta-analysis was conducted to evaluate the efficacy and safety of FMT for treatment of active UC.

**Methods:**

We searched Cochrane, Medline, Web of Science, and Embase from inception to February 2020. Randomized controlled trials (RCTs) recruiting adults with active UC, which compared FMT with controls, were eligible. The primary outcome was combined clinical remission with endoscopic remission/response. Secondary outcomes included clinical remission, endoscopic remission, and serious adverse events. Relative risk (RR) with 95% confidence interval (CI) is reported.

**Results:**

Five RCTs with 292 participants were eligible for inclusion. When data were pooled for all patients, FMT was associated with a higher combined clinical remission with endoscopic remission/response; the RR of combined outcome not achieving after FMT vs. control was 0.79 (95% CI 0.70-0.88). FMT delivered via lower gastrointestinal route was superior to upper gastrointestinal route with regard to combined clinical remission with endoscopic remission/response (RR = 0.79, 95% CI 0.70-0.89). FMT with pooled donor stool (RR = 0.69, 95% CI 0.56-0.85) and higher frequency of administration (RR = 0.76, 95% CI 0.62-0.93) may be more effective with regard to clinical remission. There was no statistically significant difference in serious adverse events with FMT compared with controls (RR = 0.98, 95% CI 0.93-1.03).

**Conclusion:**

FMT shows a promising perspective with comparable safety and favorable clinical efficacy for the treatment of active UC in the short term. However, further larger, more rigorously conducted RCTs of FMT in UC are still needed in order to resolve the controversial questions.

## 1. Introduction

Ulcerative colitis (UC) is characterized by chronic inflammation of the colon, as well as the periodicity of disease progression and remission [1]. The precise etiology of UC is unclear, which is thought to be multifactorial with the interaction of genetic susceptibility, environmental factors, gut microbiota, and dysregulated immune responses [2]. The imbalance of the gut microbiota has been suggested to markedly impact UC progression [3].

Fecal microbiota transplantation (FMT) refers to the therapeutic procedure of transplanting fecal bacteria from healthy persons into patients [4]. It is highly efficacious for the treatment of recurrent Clostridium difficile infection (CDI), with mean cure rates in the range of 87%-90% [5, 6]. Beyond CDI, FMT has been investigated as a treatment option in a variety of diseases, such as inflammatory bowel disease (IBD), irritable bowel syndrome (IBS), hepatic encephalopathy, autism, metabolic syndrome, and so on [7]. Since the first case of FMT for the treatment of UC was described by Justin Bennet in 1989 [8], there have been several case reports, case series, and randomized controlled trials (RCTs) in recent years on this topic. However, the efficacy and safety of FMT for treatment of UC is still controversial. Although there were meta-analyses examining this issue [9, 10], one of them by Narula et al. [9] did not identify the study by Crothers et al. [11] which was available in abstract form. This meant that the data of this previous meta-analysis was absence from one RCT using upper gastrointestinal tract to administer FMT. The other meta-analysis by Tang et al. [10] did not differentiate patients with active UC and UC in remission. In order to evaluate the efficacy and safety of FMT in active UC and update the previous systematic reviews, we conducted a systematic review and meta-analysis using only high-quality evidence.

## 2. Materials and Methods

### 2.1. Literature Search Strategy

A systematic retrieval of records was performed in accordance with the PRISMA statement (Preferred Reporting Items for Systematic reviews and Meta-Analyses) and Cochrane guidelines. A literature search was performed using Cochrane, Medline, Web of Science, and Embase from inception to February 2020. We also searched by hand supplementary data and relative references for potentially eligible studies. The medical literature was searched using the following terms: {FMT or [(faecal or fecal or feces or faeces or stool) and (transplant or microbiota or transfusion or implant or instillation or donor or enema or reconstitution or infusion or transfer)] or bacteriotherapy} and [UC or (ulcerative colitis)]. Both free-text words and subject headings were searched. There were no language limits.

### 2.2. Inclusion/Exclusion Criteria

Studies included in this meta-analysis were required to meet the following criteria: (1) randomized controlled trial; (2) adult subjects (participants aged ≥ 18 years) with active UC assessed by clinical scores; (3) data of clinical efficacy, including clinical remission, endoscopic remission/response, and safety of FMT available; and (4) experimental group received donor FMT, and control group received placebo or an autologous FMT. Patients receiving FMT through different delivery routes (i.e., colonoscopy, nasojejunal tube, nasogastric tube, or enemas) were all eligible. Studies were excluded if they did not provide sufficient information, including data not obtained after contacting authors.

### 2.3. Outcome Assessment

The primary outcome was combined clinical remission with endoscopic remission/response within 12 weeks after FMT. Secondary outcomes included clinical remission, endoscopic remission/response, and safety of FMT which was assessed by serious adverse events (SAEs) during FMT. SAEs during FMT were defined as subjects with adverse events requiring treatment, hospitalization, surgery, or death during FMT procedure. Subgroup analyses of different delivery routes of FMT administration, number of donors, and frequency of FMT administration were also conducted.

### 2.4. Data Extraction and Quality Assessment

Two authors (L.X. and L.Y.) carried out literature search and data extraction independently. They reviewed all articles, initially by title and abstract, then by full text, to determine whether eligibility. When multiple publications related to the same patient group, the most complete data set was included. Disagreements were resolved by consensus with the senior author (C.M.). The Cochrane's risk of bias was used to evaluate the study quality of RCTs [12]. This assessment was based on seven criteria: (1) random sequence generation (selection bias), (2) allocation concealment (selection bias), (3) blinding of participants and personnel (performance bias), (4) blinding of outcome assessment (detection bias), (5) incomplete outcome data (attrition bias), (6) selective reporting (reporting bias), and (7) other sources of bias. The risk of bias was assessed as “low,” “high,” or “unclear.” A quality score > 3 points (4-7 points) indicated a high-quality study.

### 2.5. Statistical Analysis

Data were pooled using a random-effects model, which can provide a more conservative estimate than a fixed-effects model when heterogeneity is present. The risk ratio (RR) with 95% confidence intervals (CI) was used to measure the effects in indirect comparisons, and a *P* value<0.05 was considered a statistically significant difference. We tested for heterogeneity using the chi-squared test and *I*^2^ test. The chi-squared test suggests heterogeneity between studies with a *P* < 0.10. The *I*^2^ test describes the percentage of variability in effect estimates that is due to heterogeneity rather than chance, used a cut off ≥ 50% to define a significant degree of heterogeneity [13]. For assessment of publication bias, we planned to perform funnel plots and calculated Egger's regression intercept for studies, if there were sufficient (≥10) eligible studies included in the meta-analysis [14]. Statistical analyses were performed using Review Manager Version 5.3 (RevMan for Windows 2014, the Nordic Cochrane Centre, Copenhagen, Denmark).

## 3. Results

### 3.1. Search Results and Study Characteristics

**Figure 1:**
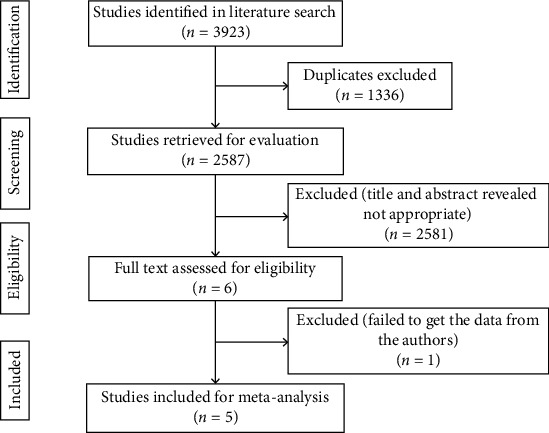
Flow diagram of search strategy.

The search strategy identified a total of 3923 citations, which included 1336 duplicates. Titles and abstracts of 2587 citations were screened, and only 6 citations were deemed potentially eligible. After reviewing the full text carefully, 1 citation was excluded, because we failed to get the data from the authors. Finally, 5 studies [11, 15–18] were eligible for the meta-analysis (Figure 1).

All 5 eligible studies with 292 participants were prospective RCTs, which included 147 patients who received donor FMT and 145 patients who received placebo or an autologous FMT. All participants were patients with mild to moderate active UC. Two trials administered FMT through the upper gastrointestinal tract (naso-duodenal infusion or oral capsules) [11, 17], and three trials administered FMT through the lower gastrointestinal tract (colonoscopy infusion and enema) [15, 16, 18]. Three trials used pooled donors' stool (2-7 donors) for FMT preparation [11, 15, 16], and two trials used single donor's stool [17, 18]. Two trials [15, 17] used low frequency of FMT infusion (2-3 times total), and three trials used higher frequency of administration (6-84 times total) [11, 16, 18]. Two trials [15, 17] compared efficacy of donor FMT with autologous FMT, and three trials [11, 16, 18] compared FMT with placebo. Participants of two trials received preantibiotic and bowel lavage pretreatment [11, 17], two trials received bowel lavage but not preantibiotic pretreatment [15, 16], and one trial did not report pretreatment information [18]. Evaluation duration of the studies was between 7 and 12 weeks. All five trials provided dichotomous data for response or nonresponse to FMT. The characteristics of the included studies are summarized in Table 1.

### 3.2. Quality Assessment and Publication Bias

**Figure 2:**
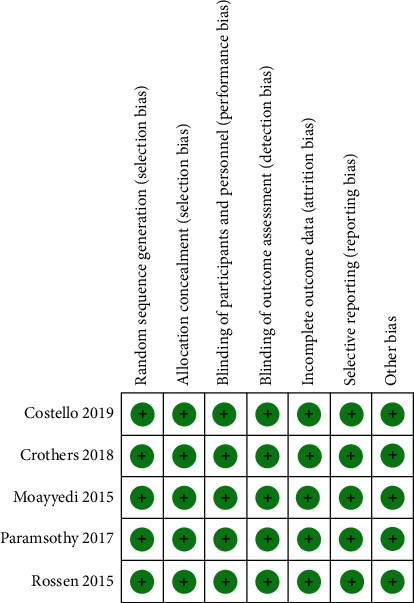
Risk of bias of included studies.

According to the Cochrane's risk of bias for assessing study quality, all studies we included were demonstrated as “high” rating (Figure 2). But there were too few studies to assess publication bias using funnel plot asymmetry.

### 3.3. Efficacy of Fecal Microbiota Transplantation in Ulcerative Colitis

#### 3.3.1. Combined Clinical Remission with Endoscopic Remission/Response

**Figure 3:**
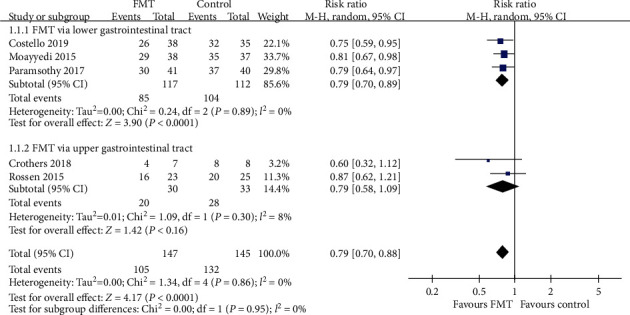
Forest plot of studies reporting combined clinical remission with endoscopic remission/response and subgroup analysis according to different delivery routes.

All five trials provided dichotomous data for response or nonresponse to FMT. When data were pooled, there were 105 (71.4%) of 147 patients assigned to the FMT group who failed to achieve combined clinical remission and endoscopic remission/response, compared with 132 (91.0%) of 145 assigned to the control group. The pooled RR of combined outcome not achieving after FMT vs. control was 0.79 (95% CI 0.70-0.88, *P* < 0.0001), with a low risk of heterogeneity detected between studies (Chi^2^ = 1.34, *I*^2^ = 0%, *P* = 0.86) (Figure 3).

**Figure 4:**
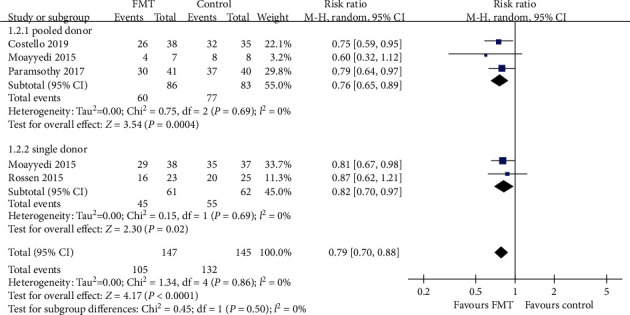
Forest plot of studies reporting combined clinical remission with endoscopic remission/response and subgroup analysis according to number of donors.

**Figure 5:**
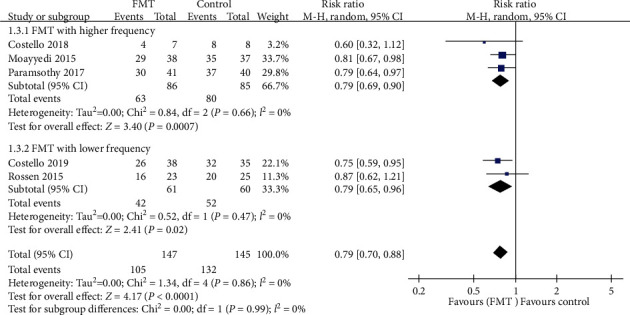
Forest plot of studies reporting combined clinical remission with endoscopic remission/response and subgroup analysis according to frequency of FMT administration.

We performed three subgroup analyses which are shown in Figures 3–5. Analysis according to the delivery route of administration demonstrated no benefit via the upper gastrointestinal tract in two pooled studies (RR = 0.79, 95% CI 0.58-1.09, Chi^2^ = 1.09, *I*^2^ = 8%, *P* = 0.30) [11, 17], but a beneficial effect when the lower gastrointestinal tract was used when data were pooled from three studies (RR = 0.79, 95% CI 0.70-0.89, Chi^2^ = 0.24, *I*^2^ = 0%, *P* = 0.89) [15, 16, 18]. When the number of donors' stools was studied, a beneficial effect was demonstrated in both pooled donor stool of three trials (RR = 0.76, 95% CI 0.65-0.89, Chi^2^ = 0.75, *I*^2^ = 0%, *P* = 0.69) [11, 15, 16] and single donor stool of two trials (RR = 0.82, 95% CI 0.70-0.97, Chi^2^ = 0.15, *I*^2^ = 0%, *P* = 0.69) [17, 18] compared with the control group. The same beneficial effect could also be seen in both higher frequency of administration of three trials (RR = 0.79, 95% CI 0.69-0.90, Chi^2^ = 0.84, *I*^2^ = 0%, *P* = 0.66) [11, 16, 18] and lower frequency of two trials (RR = 0.79, 95% CI 0.65-0.96, Chi^2^ = 0.52, *I*^2^ = 0%, *P* = 0.47) [15, 17] compared with the control group.

#### 3.3.2. Clinical Remission

**Figure 6:**
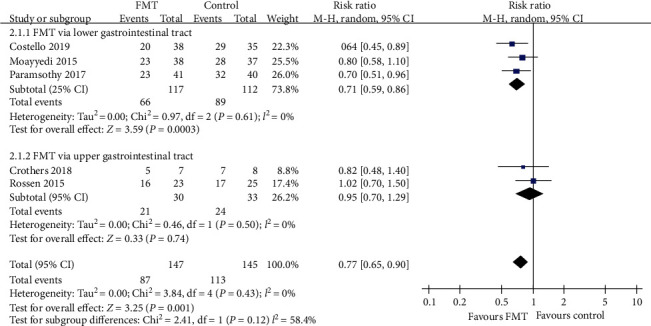
Forest plot of studies reporting clinical remission and subgroup analysis according to different delivery routes.

With regard to clinical remission, more patients receiving donor FMT achieved this outcome compared with those receiving control interventions, with the pooled RR of not achieving remission being 0.77 (95% CI 0.65-0.90, Chi^2^ = 3.84, *I*^2^ = 0%, *P* = 0.43) (Figure 6). The pooled rate of clinical remission was 40.8% (60 of 147 patients) in the FMT group and 22.1% (32 of 145 patients) in the control group.

**Figure 7:**
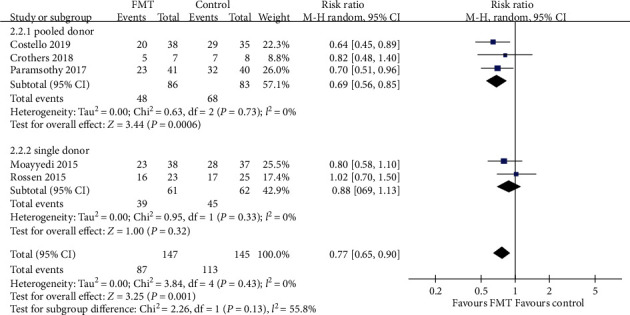
Forest plot of studies reporting clinical remission and subgroup analysis according to number of donors.

**Figure 8:**
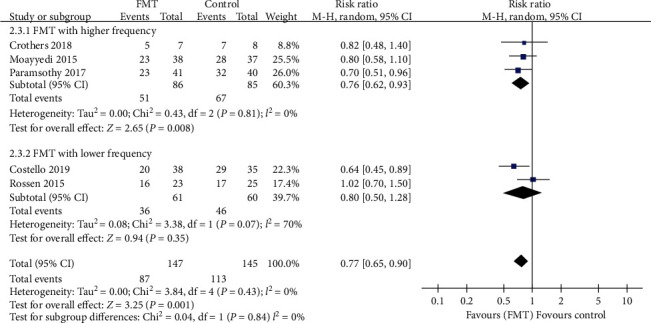
Forest plot of studies reporting clinical remission and subgroup analysis according to frequency of FMT administration.

Subgroup analyses, according to the delivery route of administration, number of donors' stools, and frequency of FMT administration, were performed, which showed a significantly beneficial effect in the lower gastrointestinal tract subgroup (RR = 0.71, 95% CI 0.59-0.86, Chi^2^ = 0.97, *I*^2^ = 0%, *P* = 0.61), pooled donor subgroup (RR = 0.69, 95% CI 0.56-0.85, Chi^2^ = 0.63, *I*^2^ = 0%, *P* = 0.73), and higher frequency of administration subgroup (RR = 0.76, 95% CI 0.62-0.93, Chi^2^ = 0.43, *I*^2^ = 0%, *P* = 0.81). But there were no significant benefits in the upper gastrointestinal tract subgroup (RR = 0.95, 95% CI 0.70-1.29, Chi^2^ = 0.46, *I*^2^ = 0%, *P* = 0.50), single donor subgroup (RR = 0.88, 95% CI 0.69-1.13, Chi^2^ = 0.95, *I*^2^ = 0%, *P* = 0.33), and lower frequency subgroup which had statistically significant heterogeneity (RR = 0.80, 95% CI 0.50-1.28, Chi^2^ = 3.38, *I*^2^ = 70%, *P* = 0.07). The three subgroup analyses data are shown in Figures 6–8.

#### 3.3.3. Endoscopic Remission

**Figure 9:**
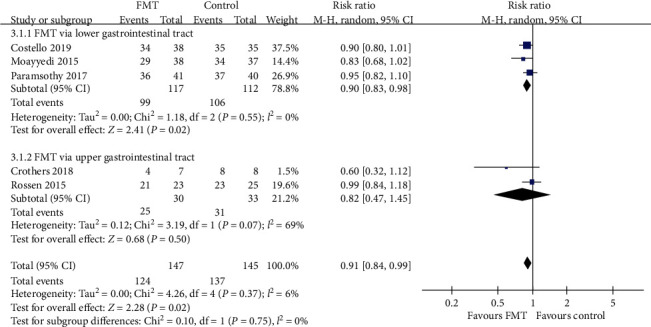
Forest plot of studies reporting endoscopic remission and subgroup analysis according to different delivery routes.

The pooled RR for not achieving endoscopic remission with donor FMT compared with controls was 0.91 (95% CI 0.84-0.99, Chi^2^ = 4.26, *I*^2^ = 6%, *P* = 0.37) (Figure 9). The pooled rate of endoscopic remission for patients who received donor FMT was 15.6% (23 of 147 patients) compared with 5.8% (8 of 137 patients) for patients in the control group.

**Figure 10:**
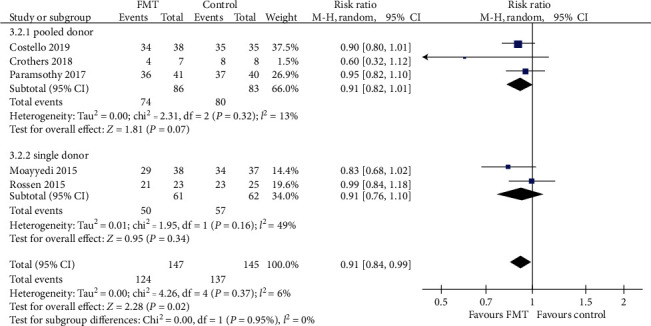
Forest plot of studies reporting endoscopic remission and subgroup analysis according to number of donors.

**Figure 11:**
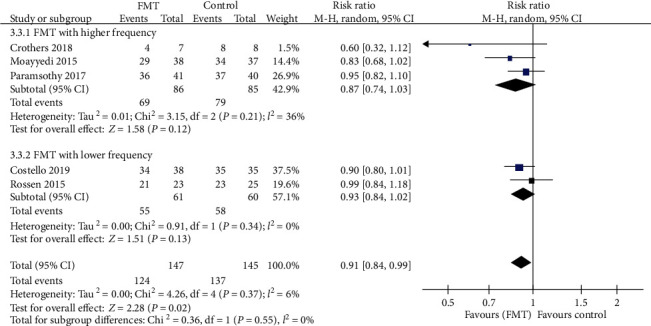
Forest plot of studies reporting endoscopic remission and subgroup analysis according to frequency of FMT administration.

Further subgroup analyses demonstrated a slightly beneficial effect in the lower gastrointestinal tract subgroup (RR = 0.90, 95% CI 0.83-0.98, Chi^2^ = 1.18, *I*^2^ = 0%, *P* = 0.55). But there were no significant benefits when the FMT group compared with the control group in the upper gastrointestinal tract subgroup (RR = 0.82, 95% CI 0.47-1.45) with statistically significant heterogeneity (Chi^2^ = 3.19, *I*^2^ = 69%, *P* = 0.07), the pooled donor subgroup (RR = 0.91, 95% CI 0.82-1.01, Chi^2^ = 2.31, *I*^2^ = 13%, *P* = 0.32), the single donor subgroup (RR = 0.91, 95% CI 0.76-1.10, Chi^2^ = 1.95, *I*^2^ = 49%, *P* = 0.16), the higher frequency subgroup (RR = 0.87, 95% CI 0.74-1.03, Chi^2^ = 3.15, *I*^2^ = 36%, *P* = 0.21), and the lower frequency subgroup (RR = 0.93, 95% CI 0.84-1.02, Chi^2^ = 0.91, *I*^2^ = 0%, *P* = 0.34). Relevant data are shown in Figures 9–11.

#### 3.3.4. Safety of FMT in UC

**Figure 12:**
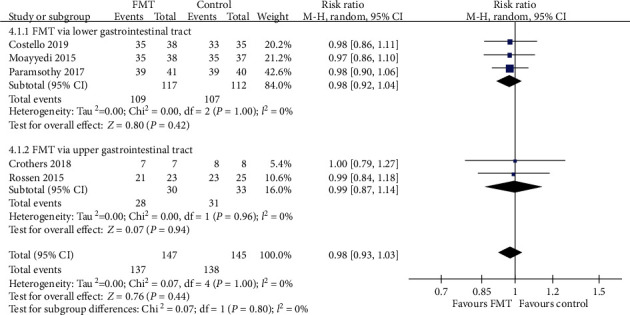
Forest plot of studies reporting serious adverse events and subgroup analysis according to different delivery routes.

**Figure 13:**
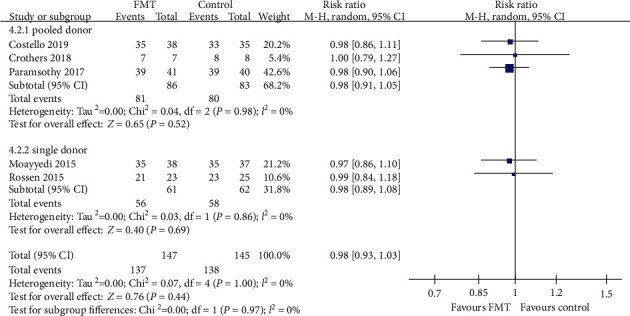
Forest plot of studies reporting serious adverse events and subgroup analysis according to number of donors.

**Figure 14:**
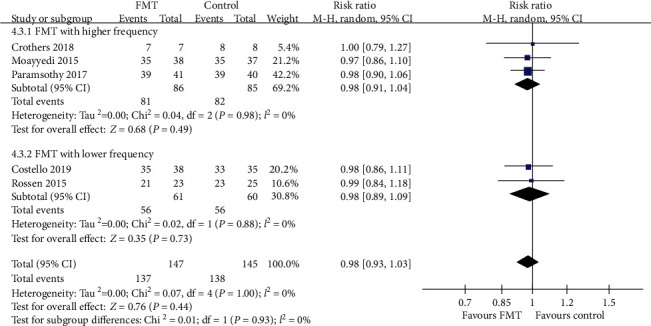
Forest plot of studies reporting serious adverse events and subgroup analysis according to frequency of FMT administration.

SAE data were provided by all of the five trials. There were no significant differences between patients receiving donor FMT compared with control patients with regard to SAEs. When data were pooled from the five RCTs, there were 10 of 147 (6.8%) patients assigned to FMT who reported SAEs, compared with 7 of 145 (4.8%) allocated to the control group. The pooled RR was 0.98 (95% CI 0.93-1.03, Chi^2^ = 0.07, *I*^2^ = 0%, *P* = 1.00) (Figure 12). Further subgroup analyses, including delivery routes, number of donors, and frequency of FMT administration, indicated no significant differences between FMT group and control group (Figures 12–14).

Individual SAEs included worsening colitis (*n* = 3) who needed admit to hospital for intravenous corticosteroid therapy or colectomy, C difficile colitis requiring colectomy (*n* = 1), pneumonia (*n* = 1), patchy inflammation of the colon and rectal abscess formation (*n* = 2), worsening abdominal discomfort tested positive for C difficile toxin (*n* = 1), small bowel perforation (*n* = 1), and abdominal pain (*n* = 1) in the FMT group. In the control group, individual SAEs included worsening colitis (*n* = 4), patchy inflammation of the colon and rectal abscess formation (*n* = 1), cytomegaloviruses (CMV) infection (*n* = 1), and cervix carcinoma (*n* = 1). Not all of them were related to FMT.

## 4. Discussion

This systematic review and meta-analysis evaluated the efficacy and safety of FMT for the treatment of active UC, synthesizing evidence from the available RCTs conducted to date. Five trials, of which one was abstract, fulfilling inclusion criteria were identified eligible. When data from all studies were pooled, there were significant improvements in the primary outcome (combined clinical remission with endoscopic remission/response) and secondary outcomes (clinical remission and endoscopic remission) when FMT vs. control. Our meta-analysis demonstrated that FMT is effective to mild to moderate active UC in the short term. Additionally, a recent pilot study showed maintenance FMT may help sustain clinical, endoscopic, and histological remission in patients with UC who are in clinical remission for a long term of 48 weeks [19], which meant FMT may also have beneficial effects in maintenance of UC.

With regard to delivery routes of FMT, our subgroup meta-analyses revealed better outcomes of lower gastrointestinal tract in both primary and secondary outcomes of FMT with controls. However, FMT via upper gastrointestinal tract did not show beneficial effects in any of the subgroup analyses when comparing FMT with controls. This result was not concordant with a recent study based on 134 UC patients, which proved no difference in efficacy between patients who received FMT from midgut and those from colonic transendoscopic enteral tubing (TET) [20]. The result also was not consistent with one of our recent prospective studies based on 9 UC patients, which showed no significant difference on the efficacy of FMT for treatment of UC between the nasojejunal tube and TET delivery routes [21]. The reason for these discordant results may be different patient inclusion criteria (mild to severely active UC patients were included in the two studies). With regard to the lower gastrointestinal route of FMT administration, the latest progress is colonic TET, which is a safe, convenient, and reliable procedure for FMT that results in a high degree of patient satisfaction [22, 23]. The experience of FMT through TET of patients with IBD leads them to maintain a positive attitude towards FMT [24]. Therefore, FMT delivery methods need to be rationally designed taking into account efficacy and recipient factors.

Another finding from our subgroup analyses was the apparently higher efficacy of pooled donor stools than single donor stools on clinical remission but not on combined clinical remission with endoscopic remission/response and endoscopic remission. The result was consistent with a previous study which suggested that remission rates of UC patients could be enhanced by pooling stools from multiple donors to increase microbial diversity [16, 25]. Other studies also revealed the efficacy of FMT in UC was related to compositional and functional differences in the donor's and recipient's gut microbiota. For example, a previous small study including 8 refractory UC patients reported that higher bacterial species richness in donors was associated with successful transplantation [26]. Another study showed that sustained remission of UC patients was associated with butyrate-producing organisms, and relapse was associated with Proteobacteria and Bacteroidetes [27]. In addition, a recent prospective study demonstrated that the differences of the recipients' relative abundance in Eggerthella, Lactobacillus, and Ruminococcus between pre-FMT and 5 days post-FMT were remarkably correlated with the long-term clinical remission [28]. As a result, selecting donors based on microbial indicators and/or capability of the donor microbiota may be important for improved FMT efficacy [7].

Similar results were shown in the third subgroup analyses which revealed a beneficial effect of higher frequency of FMT administration than lower frequency on clinical remission but not on combined clinical remission with endoscopic remission/response and endoscopic remission. However, frequency of administration and optimal overall duration is still unclear as study parameters were not directly comparable across different studies [7]. Some authors considered higher frequency of administration as a high treatment burden that would likely limit applicability to practice [15]. Further studies should evaluate parameters such as dosage frequency and total treatment duration.

When data were pooled from studies reporting SAEs, although total SAEs were more frequent among FMT patients (10 patients) than among those assigned to control group (7 patients), this difference was not statistically significant. It demonstrated that FMT is relatively safe for treatment of patients with active UC in the short term. A systematic review from this year revealed that FMT-related adverse events (AEs) were observed in 19% of FMT procedures, and diarrhea (10%) and abdominal discomfort/pain/cramping (7%) were most frequently reported. SAEs were reported in 1.4% of patients (0.99% microbiota-related SAEs), and 80% (4 of 5 patients) of FMT-related deaths were reported in patients receiving FMT via the upper gastrointestinal tract [29]. Another previous study analyzed the long-term safety of FMT in active UC with the follow-up ranged from 1 to 5 years [20]. They observed 17.4% (43/247) FMT-related AEs including one SAE. They also found that both the method of preparation of microbiota from stool using the automatic system (recently named as washed microbiota transplantation [30]) and the delivery method of colonic TET were associated with a lower rate of FMT-related AEs. All of these results demonstrated that FMT-related AEs were mild or moderate and self-limiting. However, its methodology should be improved to reduce both delivery-related AEs and microbiota-related AEs [29].

Besides the above aspects, there were likely additional factors that could contribute to the accuracy of the final results, such as the transplantation stool dosage, the frequency of administration, pretreatment antibiotics use, bowel lavage, and so on. All of these factors remain ambiguous and controversial. Washed microbiota preparation, a recent named concept based on the automatic microfiltration machine (GenFMTer, Nanjing, China), makes delivering a precise dose of the enriched microbiota feasible, instead of using the weight of stool [30]. This method may resolve the bias between studies due to differences of stool dosage in the future. Additionally, a recent prospective study demonstrated that patients with UC should undergo the second course of FMT within 4 months after the first course of FMT for maintaining the long-term clinical benefits [28]. In terms of pretreatment antibiotics use, two trials [11, 17] had antibiotic pretreatment as part of their methods, two trials [15, 16] did not adopt the use of antibiotics prior to FMT, and one trial [18] did not report this item. Although a recent study demonstrated that combination therapy of FMT and antibiotics was more effective than FMT therapy alone in restoring Bacteroidetes diversity in UC [31], antibiotic pretreatment remains controversial. The latest consensus in 2020 (Nanjing consensus on methodology of washed microbiota transplantation) stated that “Antibiotics should be stopped 12-48h before microbiota delivery” [32]. Future studies should specifically assess the role of antibiotics prior to FMT in different conditions and its cost-effectiveness [7].

Although FMT shows comparable safety and favorable clinical efficacy for the treatment of active UC in the short term, there were limitations of the included studies in our meta-analysis. All the included RCTs recruited patients with mild-moderate active UC, instead of serious conditions. However, patients with severely active UC were difficult to treat in clinic, and FMT was generally used to resolve these serious conditions. Most of patients with severely active UC were not suitable for RCT. As a result, further studies should pay more attention to these patients.

## 5. Conclusions

In conclusion, this systematic review and meta-analysis showed advantage of FMT over controls in clinical remission, endoscopic remission, and combined them together in patients with active UC when data from all RCTs were considered. In addition, the lower gastrointestinal route of delivery, pooled donor stool, and higher frequency of administration may be more effective. Meanwhile, no significant difference was noted on SAEs between FMT and the control group. Therefore, this meta-analysis demonstrated that short-term use of FMT is beneficial and safe for clinical and endoscopic improvements in patients with mild to moderate active UC. However, there have been only a few eligible RCTs conducted to date, so it is difficult to draw firm conclusions. Future RCTs are still required to address questions regarding donor selection, treatment prior to FMT, ideal stool or microbiota dosage, frequency of administration, predictors of patients most likely to respond, the most effective delivery route in different conditions, and cost-effectiveness, which remain controversial.

## Figures and Tables

**Table 1:** Characteristics of randomized controlled trials of fecal microbiota transplantation vs. control in active ulcerative colitis.

Study	Country	No. patients (FMT/control)	Severity	Donor	Delivery route	Frequency	Dosage	Preantibiotic	Bowel lavage	Control intervention	Time of evaluation	Combined clinical and endoscopic improvement	Clinical remission	Endoscopic remission/response	Combined clinical remission and endoscopic remission/response (FMT/control)	Clinical remission (FMT/control)	Endoscopic remission (FMT/control)	Serious adverse events (FMT/control)
Costello 2019	Australia	38/35	Mild to moderate (Mayo score 3-10, with endoscopic subscore ≥ 2)	Healthy volunteers (pooled 3-4 donors' stool)	1 colonoscopy, 2 enemas	3 times over 7 days	Total stool weight 100 g	No	Yes	Autologous FMT	Week 8	A total Mayo score ≤ 2, with endoscopic subscore ≤ 1	SCCAI ≤ 2	Mayo endoscopic subscore < 1	12/3	18/6	4/0	3/2
Crothers 2018	USA	7/8	Mild to moderate (Mayo score: 4-10)	Healthy volunteers with high stool butyrate (pooled 2 donors' stool)	Daily FMT capsules	Daily	Capsule 0.375 g stool per time	Yes	Yes	Placebo	Week 12	A total Mayo score < 3, with decrease in Mayo endoscopic subscore ≥ 1	Mayo score < 3	Decrease in Mayo endoscopic subscore ≥ 1	3/0	2/1	3/0	0/0
Paramsothy 2017	Australia	41/40	Mild to moderate (Mayo score: 4-10)	Healthy volunteers (pooled 3-7 donors' stool)	1 colonoscopy, 40 enemas	1 colonoscopic infusion, followed by enemas 5 days per week for 8 weeks	37.5 g stool per time, 150 ml infusion volume	No	Yes	Placebo	Week 8	A total Mayo score ≤ 2, with all Mayo subscores ≤ 1 and reduction in endoscopic subscore ≥ 1	Mayo score < 3	Mayo endoscopic subscore = 0	11/3	18/8	5/3	2/1
Rossen 2015	Netherland	23/25	Mild to moderate (SCCAI 4–11, with endoscopic subscore ≥ 1)	Healthy partners, relatives, or volunteers (single donor's stool)	2 naso-duodenal infusions	2 times at week 0 and week 3	500 ml	Yes	Yes	Autologous FMT	Week 12	SCCAI score ≤ 2, decrease in Mayo endoscopic subscore ≥ 1	SCCAI score ≤ 2	Mayo endoscopic subscore = 0	7/5	7/8	2/2	2/2
Moayyedi 2015	Canada	38/37	Mild to moderate (Mayo score ≥ 4, with endoscopic subscore ≥ 1)	Healthy volunteers (single donor's stool)	6 retention enemas	Once per week for 6 weeks	50 g, 300 ml	NR	NR	Placebo	Week 7	A full Mayo score < 3 and endoscopic subscore = 0	Mayo score < 3	Mayo endoscopic subscore = 0	9/2	15/9	9/3	3/2

NR: not reported.

## Data Availability

The retrospective data used to support the findings of this study are included within the article.

## Additional Points

*PRISMA 2009 Checklist Statement*. The authors have read the PRISMA 2009 Checklist, and the manuscript was prepared and revised according to the PRISMA 2009 Checklist.
